# Highly Transparent Anti-Smudge Coatings for Self-Cleaning, Controllable Liquid Transport, and Corrosion Resistance

**DOI:** 10.3390/polym17030302

**Published:** 2025-01-23

**Authors:** Hua Xu, Chao Chen, Shunfeng Hu, Yiqi Chen, Dengle Duan, Guowei Liang, Ximing Zhong

**Affiliations:** 1 Key Laboratory of Agricultural Green Fine Chemicals of Guangdong Higher Education Institution, School of Chemistry and Chemical Engineering, Zhongkai University of Agriculture and Engineering, Guangzhou 510225, China; dlutxuhua@163.com (H.X.); 18852922663@163.com (C.C.); hsfsgdsg@163.com (S.H.); w18938327099@163.com (Y.C.); 2 Key Laboratory of Green Processing and Intelligent Manufacturing of Lingnan Specialty Food, Ministry of Agriculture, Zhongkai University of Agriculture and Engineering, Guangzhou 510225, China; duandengle@zhku.edu.cn; 3 School of Materials Science and Engineering, South China University of Technology, Guangzhou 510641, China; lgw241331@163.com

**Keywords:** anti-smudge coatings, self-cleaning performance, anti-ink ability, corrosion resistance, mechanical robustness

## Abstract

Highly transparent anti-smudge coatings are attractive for diverse fields due to their inherent repellency against various contaminants and the ability to keep surfaces clean. In this work, a novel fluorine-free anti-smudge coating system was developed by using poly(dimethysiloxane), tris(hydroxymethyl) aminomethane, and isophorone diisocynate to synthesize a hexa-functional coating precursor and utilizing hexamethylene diisocyanate trimer as a curing agent. The resultant anti-smudge coatings are highly transparent and can be applied to various substrates. These coatings exhibit repellency against water, hexadecane, ink, pump oil, and crude oil and show self-cleaning performance in air and oily environments. Moreover, they display anti-ink ability and can be employed to reduce bacterial contamination. Of note, they can endow substrates with protection against corrosion from strong acids, strong bases, salt solutions, and even aqua regia. The developed coatings also show potential for controllable liquid transport. Moreover, these versatile coatings are mechanically robust, demonstrating tolerance against abrasion, impact, and bending and also exhibiting excellent adhesion to various substrates, indicative of their availability for widespread applications.

## 1. Introduction

Anti-smudge coatings are of significance and are highly desirable for various fields due to their unique properties of being able to repel various water- and oil-based contaminants, and such an attractive property enables them to maintain clean substrates, even under extreme conditions [1,2,3,4,5]. Therefore, they are highly available for civilian, industrial, and engineering applications. For instance, they can reduce dust deposition and accumulation on residential infrastructures and facilities, which is beneficial for urban beautification and the improvement of living environments. Moreover, applications of anti-smudge coatings to equipment endow the substrates with the ability to maintain clean surfaces, reducing costs for cleaning and maintenance. In addition, applications of these functional coatings on the inner wall of oil pipelines can reduce transport friction and improve transport efficiency. Therefore, anti-smudge coatings are useful and appealing to various fields.

In view of surface coating morphology, there are mainly two approaches for fabricating anti-smudge coatings. The first one is to construct rough surfaces with low surface energy [6,7,8]. Such a low surface energy is generally endowed by the introduction of fluorinated compounds [9,10,11]. Even though extremely high contact angles and low sliding angles can be facilely achieved in this system, the inherently delicate rough textures are susceptible to external mechanical wearing [12,13,14,15]. Also, the utilization of fluorinated compounds, especially the ones with long perfluoroalkyl chains, potentially poses detrimental effects to wildlife and the environment due to high toxicity and bioaccumulation [16,17]. In addition, the existence of micro/nano roughness is prone to scatter light and, to some extent, compromising coating transparency. Instead, the anti-smudge coatings with a smooth surface can avoid the drawbacks caused by frail, rough textures.

To prepare smooth anti-smudge coatings, the following methods are generally adopted. First, grafting low-surface-tension chains on a pre-treated substrate surface is the main approach for preparing liquid-like monolayers [18,19,20]. Of note, due to the coating’s thinness, they are vulnerable to mechanical wearing. As for slippery liquid-infused porous surfaces (SLIPS), they are generally prepared by filling the treated pores with fluorinated lubricants [21,22]. However, loss of the unbound lubricants through the pathway of evaporation or leakage exerts an adverse influence on coating properties [23,24]. Different from the above coatings, polymer-based anti-smudge coatings generally possess tunable thickness, high optical transmittance, excellent adhesion to various substrates, and firm grafting of low-surface-tension components to the coating matrix, effectively addressing the inferior transparency, weak mechanical tolerance, and the loss of anti-smudge agents, as mentioned above [3,25,26,27].

To prepare polymer-based anti-smudge coatings, low coating surface energy and heavy crosslinking should be considered. Generally, fluorinated compounds and polydimethylsiloxane (PDMS) are favorable anti-smudge agents for achieving low surface energy. Of note, compared to fluorinated compounds, PDMS becomes more appealing due to its merits of nontoxicity, chemical inertness, biocompatibility, moderate cost, and environmental friendliness [28,29,30]. Also, high crosslinking density is of importance for polymer-based anti-smudge coatings because insufficient crosslinking is inclined to bring about surface reconstruction, which will deteriorate the anti-smudge performance of coatings. In previous work, mercaptoethanol-modified castor oil was reacted with hexamethoxymethylmelamine to form heavy coating crosslinking, and the low surface energy was obtained by the addition of mono-hydroxyl-terminated PDMS [31]. However, due to the poor compatibility between PDMS and polymer matrix, once the PDMS content was increased, the coating’s transparency was significantly compromised. Therefore, an excessive PDMS content generally undermines the coating’s transparency due to macroscopic phase separation. And a coating system possessing anti-smudge performance and high transparency is highly desirable.

In this work, an anti-smudge coating system with high transparency was developed by using hydroxyl-terminated poly(dimethysiloxane) (PDMS), tris(hydroxymethyl) aminomethane (THAM), and isophorone diisocyanate (IPDI) to synthesize a hexa-functional coating precursor bearing multiple hydroxyl groups to provide adequate crosslinking sites. Hexamethylene diisocyanate trimer (HDIT) was adopted as a curing agent to react with the above coating precursor to form heavy crosslinking. The structure of the synthesized coating precursor and the complete curing of the coating were verified. The resultant coatings possessed a high optical transmittance of 98.85% and showed a fairly smooth coating surface with a roughness of 0.54 nm, owing to the great compatibility of this coating system. When the coating’s thickness reached 1.0 mm, the coating maintained visible transparency. Also, these coatings exhibited repellency against water, hexadecane, ink, pump oil, and crude oil and also protected various substrates against corrosion from a strong acid, strong base, salt solution, and even aqua regia. In addition, these anti-smudge coatings exhibited self-cleaning performance in air and oily environments and showed potential in bacterial anti-adhesion and controllable liquid transport. Moreover, these coatings demonstrated mechanical robustness against abrasion, impact, and bending and also exhibited excellent adhesion to various substrates, showing potential for widespread applications.

## 2. Materials and Methods

### 2.1. Materials

Hexamethylene diisocyanate trimer (HDIT, Bayer N3390, with an NCO content of 19.6 ± 0.3%), isophorone diisocynate (IPDI, Aladdin), and dibutyltin dilaurate (Aladdin) were used as purchased without further purification. Hydroxyl-terminated poly (dimethysiloxane) (PDMS, Foshan Silchem Trading Co., Ltd., Foshan, China), 1-methoxy-2-propyl acetate (Aladdin), and 1-methyl-2-pyrrolidinone (Aladdin) were distilled under vacuum prior to use. Tris(hydroxymethyl) aminomethane (THAM) and red oil O were supplied from Shanghai Yuanye Bio-Technology Co., Ltd., Shanghai, China. And were of analytical reagent grade. Sodium chloride (NaCl), anhydrous cupric sulfate (CuSO_4_), sodium hydroxide (NaOH), concentrated sulfuric acid (H_2_SO_4_), hydrochloric acid (HCl), nitric acid (HNO_3_), hexadecane, and methylene blue were of analytical reagent grade and procured from Tianjin Damao Chemical Reagent Factory, Tianjin, China. Luria–Bertani (LB) nutrient agar was bought from Beijing Soleibao Technology Co., Ltd., Beijing, China. Glass plates (76 × 25 × 1 mm^3^) were purchased from Yancheng Feizhou Bose Plastic Co., Ltd., Yancheng, China. Aluminum plates (150 × 76 × 0.42 mm^3^), steel plates (147 × 68 × 1 mm^3^), tin plates (120 × 25 × 0.29 mm^3^), and tile plates (200 × 146 × 10 mm^3^) were kindly provided by local suppliers.

### 2.2. Synthesis of Coating Precursor

IPDI (4.00 g) and PDMS (8.37 g) were added into a 100 mL flask and mixed thoroughly by magnetic stirring, and the flask was subsequently heated to 85 °C. Thereafter, dibutyltin dilaurate (0.02 g) and 1-methoxy-2-propyl acetate (2.00 g) were added to the flask. The reaction mixture was cooled down to room temperature after continuous reaction at 85 °C for 4 h. Afterwards, THAM (2.18 g) was dissolved in 1-methyl-2-pyrrolidinone (20.0 g) at 90 °C, and the solution was added dropwise into the above mixture with magnetic stirring, and the reaction was continued for another 30 min. At last, the flask was heated to 80 °C, and the reaction proceeded for an additional 2 h to obtain the desirable coating precursor. Herein, THAM was selected as the reactive agent to endow the coating precursor with hexa-functionality, which facilitated the formation of heavy crosslinking derived from the reaction between the coating precursor and curing agent. The chemical structure of the coating precursor can be found in Figure S1.

### 2.3. Preparation of the Anti-Smudge Coatings

The coating precursor (2.00 g), HDIT (1.01 g), and 1-methoxy-2-propyl acetate (1.00 g) were thoroughly mixed together, and the solution was cast onto various substrates, such as a glass plate, aluminum plate, steel plate, tile plate, and tin plate. The coated substrates were then horizontally placed into a drying oven at 50 °C for 30 min and then cured at 140 °C for another 3 h before further tests.

### 2.4. Characterization and Measurement

The optical transmittances of the coated glass plates were measured by a Varian CARY 300 Bio UV–visible spectrometer at a 500 nm wavelength using an uncoated glass plate as a reference. A Fourier transform infrared (FTIR) spectrum was recorded by a Tensor-27 spectrometer (Bruker Optics, Germany) to detect the consumption of the isocyanate groups from the cured coatings. The thicknesses of the coatings were determined by using a portable thick gauge (LZ-990 Kett, Japan). The ^1^H NMR spectrum of the coating precursor was obtained by using a Bruker Avance Ⅲ HD 600 spectrometer, and acetone-*d_6_* was used as a solvent. A ZEISS Merlin scanning electron microscope (SEM) and a Bruker atomic force microscopy (AFM) were used to characterize the morphology of the coating surface, and the latter was also used to measure the coating surface roughness. The chemical compositions of the coatings before and after abrasion were investigated by a Thermo Fisher Scientific ESCALAB 250Xi X-ray photoelectron spectroscopy (XPS) system. For the abrasion test, a piece of cotton fabric served as an abrasion medium, and a 200 g weight was placed onto the fabric to enhance the force of abrasion. The adhesion test was conducted according to ASTM adhesion standards. Specifically, the coating surface was first cut by a blade in a cross-cut pattern (10 × 10 lines), and the cutter spacing was ∼1 mm. A 3M Scotch tape was then firmly pressed onto the cross-cut surface and removed via peeling vertically. The sample was further examined for the signs of damage and ranked according to the ASTM protocol. An impact experiment was implemented on the coated tin plate using an impact tester (QCJ-50, Shanghai Modern Environmental Engineering Technology); the general procedure included placing the coated substrates onto the stage, elevating the impact hammer (with a weight of 1000 ± 1 g) to a height of 50 cm, and freeing the hanging hammer to induce an impact on the coating. The bacterial anti-adhesion rate was evaluated via the plate count method, and S. aureus was used as the bacterium strain [32]. In particular, the coating sample with dimensions of 2.5 × 2.5 cm^2^ was immersed in a 200 mL bacterial suspension with a concentration of about 10^6^ CFU mL^−1^, and it was incubated for 2 h at 37 °C in a shaking incubator. Thereafter, sterile cotton was used to gently swab the coating surface, and then it was placed in a tube containing 20 mL of Ringer’s solution, which was further sonicated for 1 min. Afterward, 50 μL of the above solution was spread on an LB agar plate and incubated at 37 °C overnight. The bacterial colonies were recorded, and the bacterial anti-adhesion rate was determined as the ratio of bacterial reduction to bacterial colonies corresponding to the blank control. At least five measurements were conducted for each datum, and thus, each reported datum represented the average of different measurements.

## 3. Results and Discussion

### 3.1. Structure Conformation and Coating Transparency

**Figure 1:**
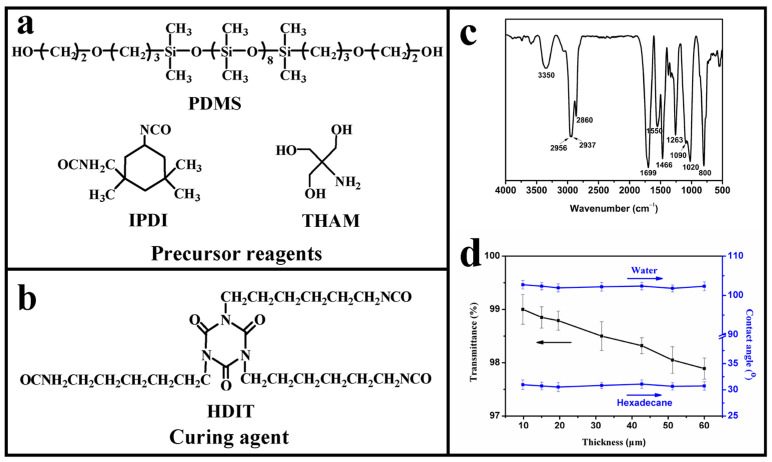
(**a**) Chemical structures of precursor agents (PDMS, IPDI, and THAM). (**b**) Chemical structures of curing agent (HDIT). (**c**) FTIR spectrum of the cured coatings. (**d**) Variations of the optical transmittance and the contact angle toward water and hexadecane on the coating surface as a function of coating thickness.

Figure 1a shows the chemical structures of PDMS, IPDI, and THAM, respectively. After the reaction between the isocyanate groups inherent in IPDI and the hydroxyl groups in PDMS, as well as the amino groups in THAM, a coating precursor with hexa-functionality was synthesized, and the chemical structure of this precursor was confirmed by ^1^H NMR characterization (seen in Figure S1). Afterward, the above coating precursor was mixed thoroughly with a curing agent (HDIT, chemical structure seen in Figure 1b) and then cast onto substrates before being subjected to thermal treatment. Considering the hexa-functionality (hydroxyl groups) of the coating precursor and the trifunctionality (isocyanate groups) of the curing agent, the reaction between the coating precursor and curing agent was expected to form heavy crosslinking. The FTIR spectrum of the cured coating is displayed in Figure 1c. Specifically, the characteristic peaks at 1090 cm^−1^, 1020 cm^−1^, and 800 cm^−1^ indicated the presence of Si-O-Si, and the characteristic peaks at 1263 cm^−1^ signified the existence of Si-CH_3_ [33]. The peak at 2956 cm^−1^ represented a -CH_3_ asymmetric stretching vibration. And the peaks at 2937 cm^−1^, 2860 cm^−1^, and 1466 cm^−1^ corresponded to the -CH_2_- asymmetric stretching vibration, symmetric stretching vibration, and bending vibration, respectively. The peak at 2265 cm^−1^, corresponding to the -NCO groups, was not found, indicating the complete consumption of isocyanate groups, and the generated carbamate groups were confirmed by the presence of the peaks at 1550 cm^−1^ (NH bending vibration), 1699 cm^−1^ (C = O stretching vibration), and 3350 cm^−1^ (NH stretching vibration). The above results confirmed the complete curing process and the successful preparation of the anti-smudge coatings.

Of note, it was found that the obtained cured coating was highly transparent. When the coating thickness was about 15 μm, the corresponding optical transmittance was 98.85%. Increasing the coating thickness insignificantly decreased the optical transmittance. Compared with previous works [4,28], the optical transmittance of the coating in this work was advantageous at the same thickness. Notably, as demonstrated in Figure S2, the coating remained visually transparent with an optical transmittance of 90.21% as the coating thickness reached 1.0 mm. Such high coating transparency was mainly attributed to the good compatibility of this coating system. Also, the smooth coating surface was conducive to the achievement of high optical transmittance. As shown in Figure S3, the coating surface is fairly smooth, and no particle, void, or wrinkle is presented. After measurement, the root-mean-square roughness of the coating surface was only 0.54 nm, far from compromising the optical transmittance. Therefore, different from the coatings with delicate rough textures that scatter light, the as-prepared anti-smudge coatings possessed high transparency.

### 3.2. Self-Cleaning Performance and Anti-Ink Ability of the Coatings

As for the anti-smudge coatings, low coating surface energy is indispensable. In this coating system, the introduction of PDMS was effective in providing the coatings with low surface energy and anti-smudge performance. And water (surface tension of ~72 mN m^−1^) and hexadecane (surface tension of ~27 mN m^−1^) were used as probe liquids for investigation. As shown in Figure 1d, the contact angle of the water was about 102.34°, and that of hexadecane was about 30.73°. This phenomenon could be justified by Young’s equation [34]. Because hexadecane possesses a lower surface tension than water, the liquids with lower surface tension are more likely to spread on the coating surface, leading to a larger contact angle of water than that of hexadecane. Also, the variations in coating thickness showed an insignificant effect on the liquid contact angle (Figure 1d). Furthermore, the sliding angle of probe liquids on the coating surface was investigated. Generally, on anti-smudge surfaces, the sliding angle decreased as the volume of liquid increased. For 5.0 μL of hexadecane, the sliding angle was about 7.60°. As the hexadecane volume increased to 20.0 μL, the sliding angle decreased to 3.81°, while 20.0 μL of water gave a sliding angle of 40.80°. When the volume of water increased to 40.0 μL, the sliding angle decreased to 18.21°. The phenomenon that the sliding angle of water was higher than that of hexadecane at the same volume was commonly presented. This was because hexadecane was miscible with PDMS, and it was able to swell PDMS chains on the coating surface, thus reducing the friction between the PDMS layer and the probe liquid [35], resulting in a smaller sliding angle than water on the coating surface.

**Figure 2:**
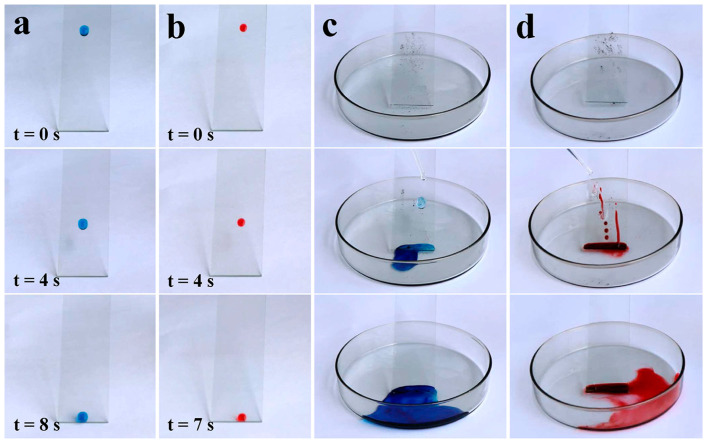
(**a**) Blue-dyed water droplet and (**b**) red-dyed hexadecane droplet sliding off the coated glass plate, respectively. Dirt scattered on the coated glass plate and cleaned with (**c**) water and (**d**) hexadecane, respectively.

Also, these anti-smudge coatings exhibited self-cleaning performance. As seen in Figure 2a, a water droplet (dyed blue) readily slid off from a coated glass plate without leaving any traces, while a water droplet wetted the uncoated one, left noticeable traces, and finally pinned on the substrate during its movements (Figure S4a). Similarly, as demonstrated in Figure 2b, a hexadecane droplet (dyed red) glided down smoothly on the coating surface with no trace left along its path. However, on the uncoated one, hexadecane spread and left a conspicuous, red-dyed path (Figure S4b). Moreover, dirt on the coating surface could also be cleaned up by probe liquids to further demonstrate self-cleaning performance. Thus, a dirt-removal test was further carried out. As seen in Figure 2c, water was dropped to remove the dirt scattered on the coating surface, and the dirt was cleaned up along with the water’s movement. Contrarily, water spread and wetted the uncoated surface, finally leaving visible dirty traces (Figure S4c). Similarly, the dirt that was scattered on the coated glass plate was carried away by hexadecane (Figure 2d), while hexadecane wetted the dirt and left noticeable traces on the untreated surface (Figure S4d). The above results indicated that self-cleaning performance could be achieved on the coating surface.

**Figure 3:**
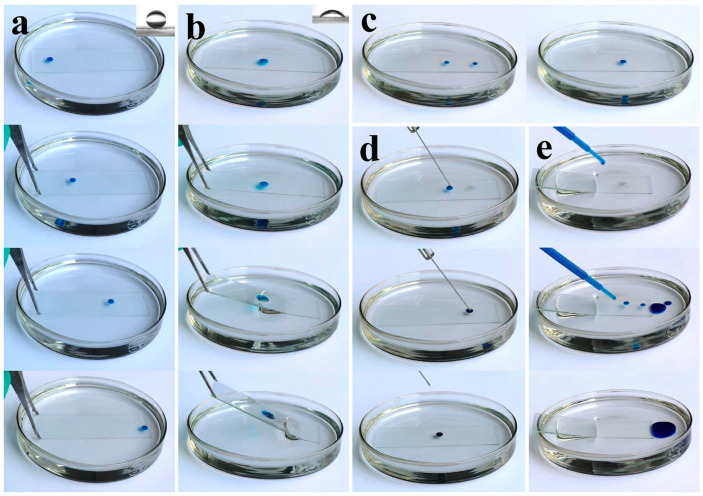
(**a**) Water droplet sitting in oil (hexadecane) and readily gliding away as the coated glass plate was slightly tilted. The inset indicated that the water droplet had a contact angle of 160.27° in oil. (**b**) Water droplets spreading on the uncoated glass plate (with a contact angle of 59.23°, as demonstrated in the inset) and pinning tightly. (**c**) Two water droplets merged into a bigger droplet on the coated glass plate in oil. (**d**) Controllable cleaning with water droplets by a needle. (**e**) Dirt being carried away by dropping water droplets in oil.

Apart from the self-cleaning performance exhibited in the air, we found that this coating also shows self-cleaning performance in an oily environment. Because of the significant polarity difference between water and oil, when immersed in oil, water droplets tend to contract maximumly, forming a spherical shape to reduce its surface-free energy. However, uncoated glass is so hydrophilic that it definitely shows more affinity to water than oil, leading to a low contact angle (59.23°; see the inset in Figure 3b) and persistent pinning of water on a pristine glass plate when it is immersed in oil (Figure 3b). In sharp contrast, water droplets formed “marble” (with a contact angle of 160.27°; see the inset in Figure 3a) on coated glass immersed in hexadecane and then readily rolled off when the coating was slightly tilted (Figure 3a; the tilting angle was about 3.0°). Consequently, on these anti-smudge coating surfaces, superhydrophobicity could be achieved in oil. Owing to the non-wetting of the coating surface toward the water in oil, two water droplets (20.0 μL) could be easily merged together and moved controllably (Figure 3c). Therefore, on coating surfaces, controllable cleaning of dirt could be achieved in oil by moving the water droplets. As shown in Figure 3d, a needle was used to control the movement of the water droplet (40.0 μL), and the dirt that was scattered on the coating surface was gradually removed as it came into contact with water. Moreover, it is worth noting that dirt on the coating surface in oil could also be removed by dropping water droplets. As demonstrated in Figure 3e, continuous water droplets readily rolled off and easily carried away the dirt scattered on a tilted coating immersed in oil, leaving no trace along the water movement paths on the coating surface. Therefore, the coatings showed potential application in self-cleaning, even in oily environments.

**Figure 4:**
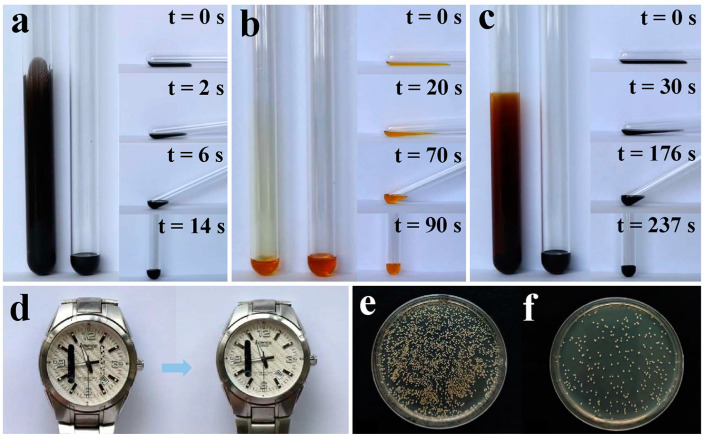
(**a**) Water-soluble black ink, (**b**) pump oil, and (**c**) crude oil, respectively, on the uncoated glass tube and the final status after application on the coated one. Photographs on the right side were used to compare the repellency of the coatings against the above contaminants. (**d**) Marker ink (oil-based) traces are left on the uncoated side of a half-coated wristwatch, while ink contracted on the coated side and can easily be removed by wiping with a tissue. (**e**) Bacterial colonies corresponding to uncoated blank control. (**f**) Bacterial colonies corresponding to the anti-smudge coatings.

Except for water and hexadecane, as aforementioned, these anti-smudge coatings showed repellency against various contaminants, including water-soluble black ink (Figure 4a), pump oil (Figure 4b), and crude oil (Figure 4c). The above contaminants were, respectively, added into the untreated glass tubes and that with inner walls coated for comparison. To form a uniform contaminant film on the wall surface, the process included lying down the test tubes, rotating them, and then returning the tubes to a vertical position. As seen in Figure 4a-c (left side), on the pristine test tubes, persistent contaminant films conspicuously remained and showed no sign of contraction, while no noticeable traces were finally left on the coated ones because of the anti-smudge performance. To further visually compare the ability of these coatings to repel black ink, pump oil, and crude oil, as demonstrated in Figure 4a–c (right side), coated tubes with the above contaminants settling on the bottom were laid down horizontally and gradually lifted from one side until reaching the vertical position so as to make the diffused contaminants backtrack. It is noteworthy that water-soluble black ink showed the shortest diffused distance and backtracking time than that of pump oil and crude oil because of its high surface tension and lower viscosity. Compared with pump oil, crude oil exhibited a longer backtracking time due to its complicated components and high viscosity. Although they displayed different contraction behaviors, no trace was finally left on the contaminated coating surface, indicating that these anti-smudge coatings exhibited repellency against water-soluble ink, pump oil, and crude oil. Therefore, they showed potential for protecting substrates from various contaminants to maintain clean surfaces and were even conducive to reducing the friction and sludge deposition of crude oil in transport pipelines.

Actually, a high contact angle and the smooth sliding of liquids do not really imply that the coatings possess anti-smudge properties because the achievement of anti-ink ability on the coating surface is more challenging. Therefore, ink (oil-based) repellency was further used to verify the anti-smudge properties of these coatings. As seen in Figure 4d, oil-based marker ink readily left a uniform and distinct black trace on the uncoated side of a half-coated glass (on a wristwatch), whereas, on the coated side, such ink readily shrunk into faint patchy markings and was easily removed by wiping with a tissue. Therefore, these anti-smudge coatings not only helped to inhibit ink deposition but also contributed to ink removal. Moreover, we found that these coatings could be applied onto various substrates without compromising the anti-smudge properties. As demonstrated in Figure S5, various coated substrates, such as steel plate, aluminum plate, and tile plate, had repellency against hexadecane and showed self-cleaning performance, and they also exhibited anti-ink ability, as demonstrated in Figure S6. The above results indicate that these coatings could be applied onto diverse substrates and bestowed them with anti-smudge properties.

As we know, bacteria are ubiquitous in both living and working environments. Considering the anti-smudge properties of these coatings, they might show repellency against bacteria and thus achieve the inhibition of bacterial adhesion. Although many superhydrophobic coatings were reported to exhibit low bacterial adhesivity through maximumly reducing the contact area of bacterial suspension and coating surface, bacteria were inclined to adhere to the defective areas due to the vulnerability of the delicate rough textures, thus causing failure in anti-bacteria properties [36]. Owing to the surface smoothness and low surface energy, the drawbacks of superhydrophobic coatings could be avoided in this coating system. Of note, the results revealed that the bacterial colonies corresponding to anti-smudge coatings were greatly reduced compared to the uncoated control blank, and the bacterial anti-adhesion rate was calculated at about 89%. Therefore, these anti-smudge coatings could be applied to various substrates to reduce bacterial contamination.

### 3.3. Controllable Liquid Transport on Coating Surface

**Figure 5:**
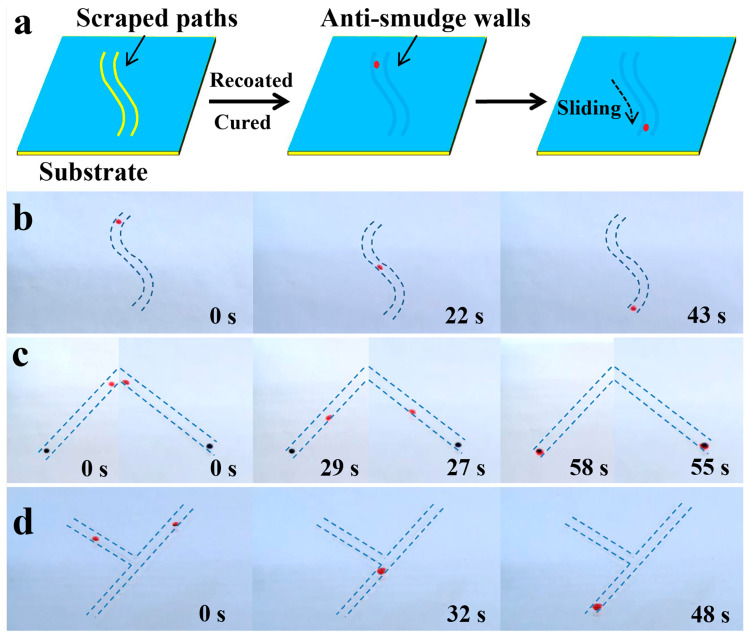
Controllable liquid transport on the coating surface. (**a**) Schematic illustration of the approach to constructing sliding paths for controllable liquid transport on the coating surface. For interpretation, visually, the yellow represents uncoated regions and substrate, while the blue represents the coated regions, and the red is on behalf of the liquid droplet applied. (**b**) A hexadecane droplet slides along a curved pattern. (**c**) A hexadecane droplet directionally glides to the target droplet on the lower left or right. (**d**) Two droplets sliding along different patterns to merge. The tilting angle was about 17.0°. The path distance for liquid sliding was about 4 mm.

As demonstrated above, liquid droplets could readily glide down without leaving any traces on an anti-smudge coating surface. Benefiting from the repellency against various liquids, we found that liquid transport could be easily controlled on the coating surface, as the sliding paths were appropriately constructed. As illustrated in Figure 5a, sliding paths could be constructed by scraping off the coating on a coated substrate with a sharp scalpel to form narrow “ravines” (i.e., uncoated paths), and then abundant anti-smudge coating solution was used to fill the uncoated paths. After curing the coating solution applied, artificial anti-smudge “walls” were built. Therefore, liquids could controllably slide along the created paths instead of gliding down perpendicularly. As expected, as seen in Figure 5b, a hexadecane droplet (about 2.0 μL, dyed red) smoothly glided down along a curved path at a tilting angle of 17.0°. Furthermore, targeted liquid transport could also be achieved. As demonstrated in Figure 5c, liquid directionally slid to the target droplet on the lower left or right. Moreover, by controllable transport, two liquid droplets could also merge into a larger one during the sliding process and glide down along the constructed path without leaving traces (Figure 5d). Therefore, these anti-smudge coatings show potential application in controllable liquid transport.

### 3.4. Corrosion Resistance of the Coatings

**Figure 6:**
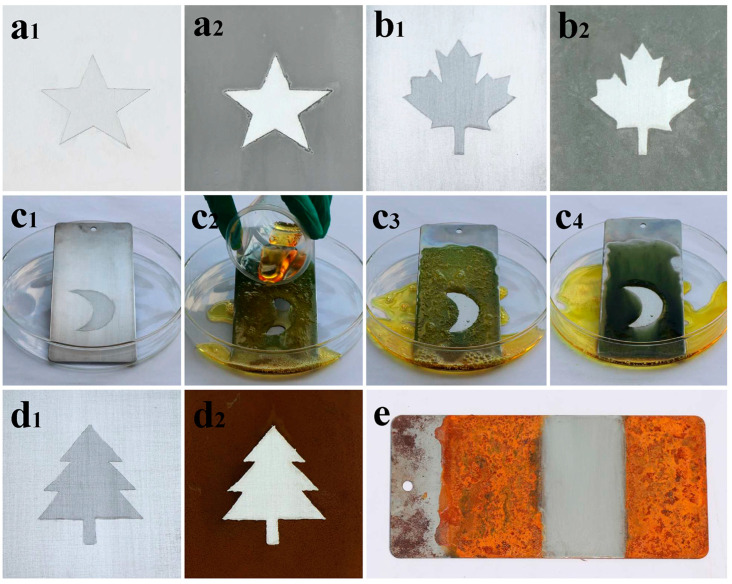
Corrosion resistance tests. (**a_1_**,**a_2_**) Photographs of an aluminum plate with a coated star-shaped pattern before and after being treated with 1.0 M NaOH solution. (**b_1_**,**b_2_**) Images of a steel plate with a coated maple leaf pattern before and after being treated with 1.0 M H_2_SO_4_ solution. (**c_1_**–**c_4_**) Snapshots of a steel plate with a coated half-moon pattern treated with aqua regia. (**d_1_**,**d_2_**) Comparison of a steel plate with a coated tree pattern before and after treated with 0.5 M CuSO_4_ solution. (**e**) A steel plate bearing coated (middle) and uncoated sections after immersion in a 3.5 wt% NaCl solution for 168 h.

Aside from exhibiting repellency against contamination from water and various organic liquids, anti-smudge coatings also perform protection against corrosion from strong acids, strong bases, and salt solutions. To evaluate the alkali-resistant ability of these anti-smudge coatings, as seen in Figure 6a_1_, an aluminum plate with a coated star-shape pattern was immersed into a strong base solution (1.0 M NaOH solution). After 5 s of immersion, bubbles began to generate on the uncoated surface due to the dissolution of surficial aluminum oxide and the subsequent reaction of the resurfaced aluminum with a base solution. As the alkali corrosion deepened, more and more bubbles appeared. After 15 min of immersion, uncoated regions were covered with a layer of resultant sediment, whereas no sign of damage was found on the coated pattern (Figure 6a_2_), indicative of the alkali-tolerant property of these coatings.

In addition, strong acid was further used to evaluate the protective properties of these anti-smudge coatings. A polished steel plate with a coated maple leaf pattern (Figure 6b_1_) was immersed into a 1.0 M H_2_SO_4_ solution. Bubbles gradually formed and became vigorous as the corrosion proceeded. After 15 min of immersion, uncoated regions were corroded severely, whereas the coated pattern showed no evidence of corrosion (Figure 6b_2_). Moreover, aqua regia (a solution mixed with highly hydrochloric acid (HCl) and nitric acid (HNO_3_) with a volume ratio of 3:1), a strongly acidic and potent oxidizing agent, was further employed to evaluate the harsh chemical corrosion resistance of these anti-smudge coatings. As demonstrated in Figure 6c_1_–c_4_, a steel plate with a coated pattern tilted against a watch glass was poured with aqua regia. On uncoated regions, aqua regia readily spread, simultaneously causing severe corrosion along with vigorous bubbles, while aqua regia obviously contracted and slid away the coated surface. As seen in Figure 6c_3_,c_4_, a small aqua regia droplet was left on the coating surface because its negligible volume was not enough to slide off, but it caused little damage to the substrate due to the protection against exposure to chemical corrosion provided by these anti-smudge coatings.

Furthermore, the prevention of anti-smudge coatings against salt solution was also investigated. A steel plate coated with a tree pattern (Figure 6d_1_) was immersed in a 0.5 M CuSO_4_ solution. Gradually, uncoated regions of the steel plate were covered with a layer of copper that precipitated out, driven by a single-displacement reaction (Figure 6d_2_), while the coated pattern showed no evidence of damage and remained clean. Moreover, a NaCl solution was further employed to investigate the protection of the coatings against a salt solution. A steel plate bearing coated and uncoated sections was immersed in a 3.5 wt% NaCl solution for 168 h. As demonstrated in Figure 6e, rust had severely developed on the uncoated sides, while little rust or damage was found on the coating surface.

The corrosion resistance of these coatings against strong acids, strong bases, various salt solutions, and even aqua regia could be attributed to the high PDMS content on the coating surface and the heavy crosslinked polymer matrix. The introduction of PDMS provides the coatings with low surface energy, leading to high repellency against various liquids, and its chemical inertness protects the coatings from exposure to harsh chemical conditions without being corroded. Moreover, a high crosslinking density results in the impermeability of the coatings, which essentially suppresses surface reconstruction and further enhances amphiphobicity, making persistent corrosion resistance possible.

### 3.5. Mechanical Robustness of the Coatings

Generally, anti-smudge surfaces with micro/nanoscale roughness are mechanically weak because of the presence of delicate rough textures that are vulnerable to external wearing. Although the smooth anti-smudge coatings prepared in this work did not exhibit the exceptional high contact angles that are available for rough surfaces, they overcame the challenge of mechanical weakness that plagued the rough ones. Due to the heavy crosslinking and the absence of frail structures, these anti-smudge coatings exhibited mechanical robustness.

**Figure 7:**
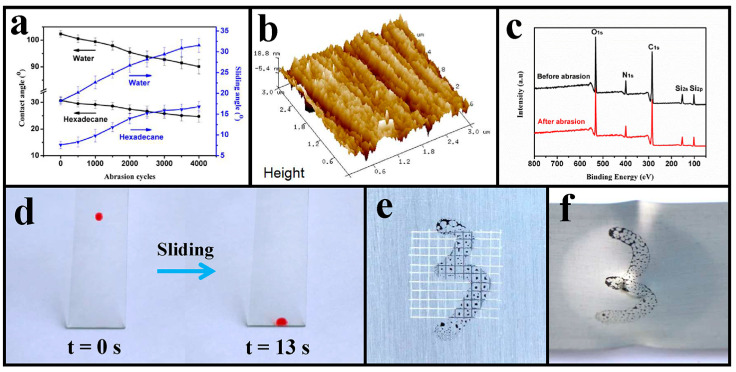
Mechanical robustness of these anti-smudge coatings. (**a**) Variations in the contact angles and sliding angles toward water and hexadecane as a function of abrasion cycles. (**b**) Three-dimensional topography image of the coating surface after suffering from 4000 abrasion cycles, and the resultant RMS roughness was 6.41 nm. (**c**) XPS analysis of the coating surface before and after 4000 abrasion cycles. (**d**) Sliding of a hexadecane droplet on the coating surface subjected to 4000 abrasion cycles. (**e**) Adhesion test on a coated tin plate. (**f**) Impact test (front side) on a coated tin plate.

To investigate mechanical robustness, the coating was first subjected to an abrasion test. As demonstrated in Figure 7a, contact angles toward water and hexadecane on the coating surface decreased along with the increase of abrasion cycles, while the sliding angles exhibited the opposite tendency. After 4000 abrasion cycles, the root-mean-square roughness of the coating surface increased from 0.54 nm to 6.41 nm (characterized via AFM; Figure 7b), while the optical transmittance of the worn coating remained 97.90% after measurement. From the XPS analysis (Figure 7c), the Si content slightly decreased from 10.15% to 9.69% on the worn surface (Table S1), leading to a decrease in contact angles and an increase in sliding angles. And the sliding of the hexadecane droplet on the coating surface that was subjected to 4000 abrasion cycles is visually demonstrated in Figure 7d. Obviously, such hexadecane droplets could readily glide down the worn coating surface without leaving any noticeable traces.

Furthermore, a standard ASTM adhesion test was used to investigate the adhesion of these coatings to substrates. The typical process included cutting the coating with a sharp blade to form a grid-like pattern, pressing 3M Scotch tape firmly onto the cross-cut surface, and quickly peeling off the tape. The fraction of the cross-cut coating removed by tape was utilized to determine the degree of adhesion. As shown in Figure 7e, although being subjected to an adhesion test, the coating exhibited no evidence of coating loss, indicating that the coatings adhered tightly to substrates with a top adhesion ranking of 5B, and such an adhesion test had little influence on the anti-ink ability. Moreover, an impact experiment was further implemented on the coated tin plate to evaluate the adhesion of the coating. As seen in Figure 7f and Figure S7a, although suffering from extreme impact damage, the coating showed no noticeable rupture on the impact sites (including the front side and opposite side), indicating the excellent adhesion of the coating on the substrate. And the impact test showed little effect on the anti-ink ability of the coating. At last, as seen in Figure S7b, even after being severely bent, the coating showed no signs of cracking and remained strongly attached to the tin plate, again confirming the excellent adhesion of the coating. Therefore, these anti-smudge coatings are mechanically robust, and they demonstrated excellent tolerance against 4000 abrasion cycles, adhesion tests, impact experiments, and bending tests, showing potential for practical applications.

## 4. Conclusions

In summary, a novel fluorine-free anti-smudge coating system was proposed and developed by using PDMS, THAM, and IPDI to synthesize a hexa-functional coating precursor to provide adequate crosslinking sites and adopting HDIT as a curing agent to react with coating precursor to form heavy crosslinking. The resultant coatings were highly transparent, and the coating surfaces were fairly smooth. These coatings exhibited repellence against various liquids and also showed self-cleaning performance in air and in oily environments. Also, they exhibited the capability to inhibit bacterial adhesion and thus could be favorably employed to reduce bacterial contamination for various substrates. In addition, they could be potentially used for controllable liquid transport. Moreover, these coatings could be applied onto different substrates, providing them with protection against various corrosive agents. Of note, they exhibited mechanical robustness and demonstrated excellent adhesion. Therefore, these newly developed anti-smudge coatings are versatile and show potential for practical applications.

## Data Availability

The original contributions presented in this study are included in the article. Further inquiries can be directed to the corresponding author.

## Supplementary Materials

The following supporting information can be downloaded at: https://www.mdpi.com/article/10.3390/polym17030302/s1, Figure S1: ^1^H NMR spectrum of the as-prepared precursor; Figure S2: The cured anti-smudge coating was visibly transparent, and even the coating thickness reached about 1.0 mm; Figure S3: SEM micrograph and AFM 3D topography image of the coating surface; Figure S4: Water droplet and hexadecane droplet spreading on the uncoated glass plate, dirt was scattered on the uncoated glass plate, leaving visible traces; Figure S5: Hexadecane droplets sliding off various coated substrates; Figure S6: Anti-ink ability demonstrated on various coated substrates; Figure S7: Opposite side of a coated tin plate after suffering from an impact experiment, and the hotography of a coated tin plate subjected to bending. Table S1: Atomic percentages of the coating surfaces before and after the abrasion test.

## References

[1] Han Y., Liu Y., Elsharkawy E.R., El-Bahy S.M., Jing D., Wu Z., Ren J., El-Bahy Z.M., Guo Z. (2024). Exploring NP-GLIDE coatings: A leap forward in the innovation of omniphobic surfaces. React. Funct. Polym..

[2] Shum R.L., Liu G. (2025). Smudge-resistant antimicrobial ladder-like polysilsesquioxane coatings. Prog. Org. Coat..

[3] Zhong X., Hu H., Yang L., Sheng J., Fu H. (2019). Robust hyperbranched polyester-based anti-smudge coatings for self-cleaning, anti-graffiti, and chemical shielding. ACS Appl. Mater. Interfaces.

[4] Zhong X., Zhou M., Wang S., Fu H. (2020). Preparation of water-borne non-fluorinated anti-smudge surfaces and their applications. Prog. Org. Coat..

[5] Zhai X., Mo Z., Yan Q., Zhao Y., Wang B., Zhang K. (2024). Anti-Smudge Organic Afterglow Panels. Adv. Funct. Mater..

[6] Jarad N.A., Imran H., Imani S.M., Didar T.F., Soeymani L. (2022). Fabrication of superamphiphobic surfaces via spray coating; a review. Adv. Mater. Technol..

[7] Li J., Li X., Liu J., Hu B., Cao X., Wu Z., Li B. (2024). Robust, breathable and antibacterial superamphiphobic cotton fabrics prepared by layer-by-layer dip coating. Appl. Surf. Sci..

[8] Wei R., Qi Y., Zhang J., Wang Y., Gao N., Lv N., Feng S., Song W., Fu A., Zhang Q. (2024). Robust and Anti-Corrosive superamphiphobic Coatings Regulated by Self-Levelling. Surf. Interfaces.

[9] Wei J., Li B., Tian N., Zhang J., Liang W., Zhang J. (2022). Scalable robust superamphiphobic coatings enabled by self-similar structure, protective micro-skeleton, and adhesive for practical anti-icing of high-voltage transmission tower. Adv. Funct. Mater..

[10] Peng J., Yuan S., Geng H., Zhang X., Zhang M., Xu F., Lin D., Gao Y., Wang H. (2022). Robust and multifunctional superamphiphobic coating toward effective anti-adhesion. Chem. Eng. J..

[11] Xia Y., Gu W., Shao L., Jiao X., Ji Y., Deng W., Yu X., Zhang Y., Zhang Y. (2023). Flexibility and abrasion tolerance of superamphiphobic coatings with rigid core-shell particles. Chem. Eng. J..

[12] Lu Y., Sathasivam S., Song J., Crick C.R., Carmalt C.J., Parkin I.P. (2015). Robust self-cleaning surfaces that function when exposed to either air or oil. Science.

[13] Yu Z.P., Kang L., Song Y.Y., Xue X.L., Liu Y., Zhang Y.F. (2024). Robust superamphiphobic coating for oil repellency and other versatility prepared via one-step spraying. Surf. Interfaces.

[14] Kota A.K., Choi W., Tuteja A. (2013). Superomniphobic surfaces: Design and durability. MRS Bull..

[15] Verho T., Bower C., Andrew P., Franssila S., Ikkala O., Ras R.H.A. (2011). Mechanically durable superhydrophobic surfaces. Adv. Mater..

[16] Jahura F.T., Mazumder N.U.S., Hossain M.T., Kasebi A., Girase A., Ormond R.B. (2024). Exploring the Prospects and Challenges of Fluorine-Free Firefighting Foams (F3) as Alternatives to Aqueous Film-Forming Foams (AFFF): A Review. ACS Omega.

[17] Li S., Huang J., Chen Z., Chen G., Lai Y. (2017). A review on special wettability textiles: Theoretical models, fabrication technologies and multifunctional applications. J. Mater. Chem. A.

[18] Cheng D.F., Urata C., Yagihashi M., Hozumi A. (2012). A statically oleophilic but dynamically oleophobic smooth nonperfluorinated surface. Angew. Chem. Int. Ed..

[19] Wang L., McCarthy T.J. (2016). Covalently attached liquids: Instant omniphobic surfaces with unprecedented repellency. Angew. Chem. Int. Ed..

[20] Macoretta D., Rabnawaz M., Grozea C.M., Liu G., Wang Y., Crumblehulme A., Wyer M. (2014). Clear antismudge unimolecular coatings of diblock copolymers on glass plates. ACS Appl. Mater. Interfaces.

[21] Wong T.S., Kang S.H., Tang S.K., Smythe E.J., Hatton B.D., Grinthal A., Aizenberg J. (2011). Bioinspired self-repairing slippery surfaces with pressure-stable omniphobicity. Nature.

[22] Li J., Zhou Z., Jiao X., Guo Z., Fu H. (2024). Bioinspired lubricant-infused porous surfaces: A review on principle, fabrication, and applications. Surf. Interfaces.

[23] Gao Z., Xu T., Miao X., Lu J., Zhu X., Song Y., Ren G., Jia Y., Li X. (2021). A thermal-driven self-replenishing slippery coating. Surf. Interfaces.

[24] Kasapgil E., Erbil H.Y., Sakir I.A. (2022). Multi-liquid repellent, fluorine-free, heat stable SLIPS via layer-by-layer assembly. Colloid. Surf. A.

[25] Rabnawaz M., Liu G. (2015). Graft-copolymer-based approach to clear, durable, and anti-smudge polyurethane coatings. Angew. Chem. Int. Ed..

[26] Huang S., Liu G., Hu H., Wang J., Zhang K., Buddingh J. (2018). Water-based anti-smudge NP-GLIDE polyurethane coatings. Chem. Eng. J..

[27] Zhu J., Fan H., Wan J. (2024). Solvent-free and UV-cured epoxy silicone coating with excellent wear resistance and antismudge properties. ACS Appl. Mater. Interfaces.

[28] Zhong X., Sheng J., Fu H. (2018). A novel UV/sunlight-curable anti-smudge coating system for various substrates. Chem. Eng. J..

[29] Lipomi D.J., Vosgueritchian M., Tee B.C.K., Hellstrom S.L., Lee J.A., Fox C.H., Bao Z. (2011). Skin-like Pressure and Strain Sensors Based on Transparent Elastic Films of Carbon Nanotubes. Nat. Nanotechnol..

[30] Yan J., Jeong Y.G. (2014). Multiwalled Carbon Nanotube/Polydimethylsiloxane Composite Films as High Performance Flexible Electric Heating Elements. Appl. Phys. Lett..

[31] Zhong X., Lv L., Hu H., Jiang X., Fu H. (2020). Bio-based coatings with liquid repellency for various applications. Chem. Eng. J..

[32] Fu Y., Jiang J., Zhang Q., Zhan X., Chen F. (2017). Robust liquid-repellent coatings based on polymer nanoparticles with excellent self-cleaning and antibacterial performances. J. Mater. Chem. A.

[33] Zhong X., Hu H., Fu H. (2018). Self-cleaning, chemically stable, reshapeable, highly conductive nanocomposites for electrical circuits and flexible electronic devices. ACS Appl. Mater. Interfaces.

[34] Atkins P. (1998). Physical Chemistry.

[35] Hu H., Liu G., Wang J. (2016). Clear and durable epoxy coatings that exhibit dynamic omniphobicity. Adv. Mater. Interfaces.

[36] Chen Y., Ao J., Zhang J., Gao J., Hao L., Jiang R., Zhang Z., Liu Z., Zhao J., Ren L. (2023). Bioinspired superhydrophobic surfaces, inhibiting or promoting microbial contamination?. Mater. Today.

