# Supplementary material for: Highly Transparent Anti-Smudge Coatings for Self-Cleaning, Controllable Liquid Transport, and Corrosion Resistance
Source: Polymers (Basel). 2025 Jan 23;17(3):302. doi: 10.3390/polym17030302 (PMC11820985; doi:10.3390/polym17030302)
Supplement: Supplementary file 1 [file polymers-17-00302-s001.zip › polymers-3436978-supplementary.pdf]

Supporting information

Highly Transparent Anti-Smudge Coatings for Self-Cleaning, Controllable Liquid Transport, and Corrosion Resistance

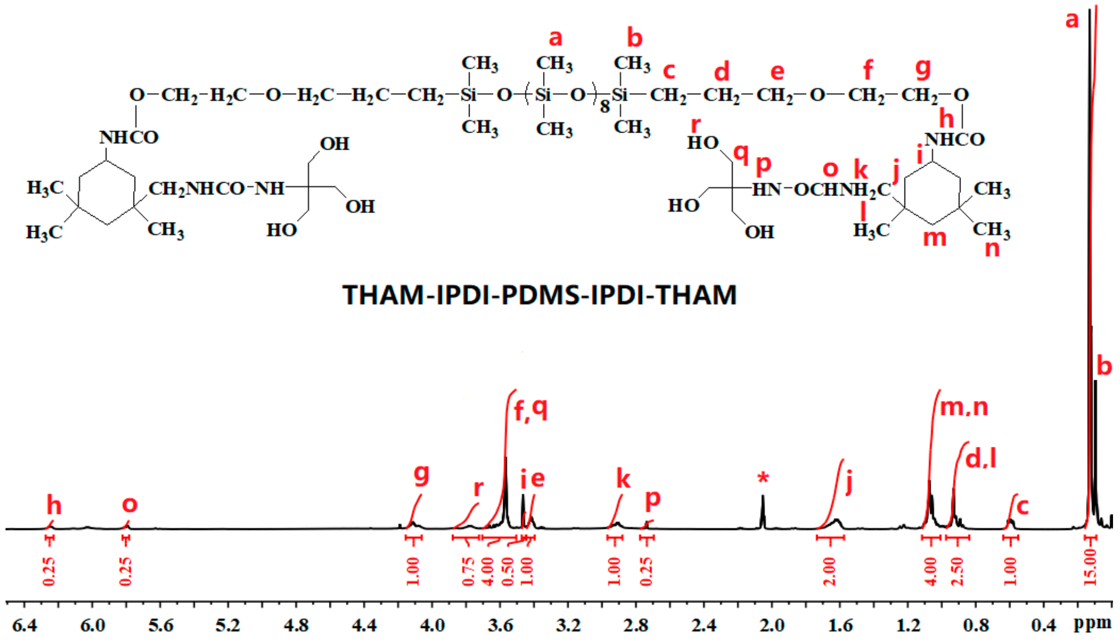

Figure S1. ^{1}H NMR spectrum of the as-prepared precursor (recorded in acetone-d_6 at 600 MHz at 298 K). Corresponding signal assignments are showed above, and the asterisked peak belongs to residual solvent peak from acetone-d_6.

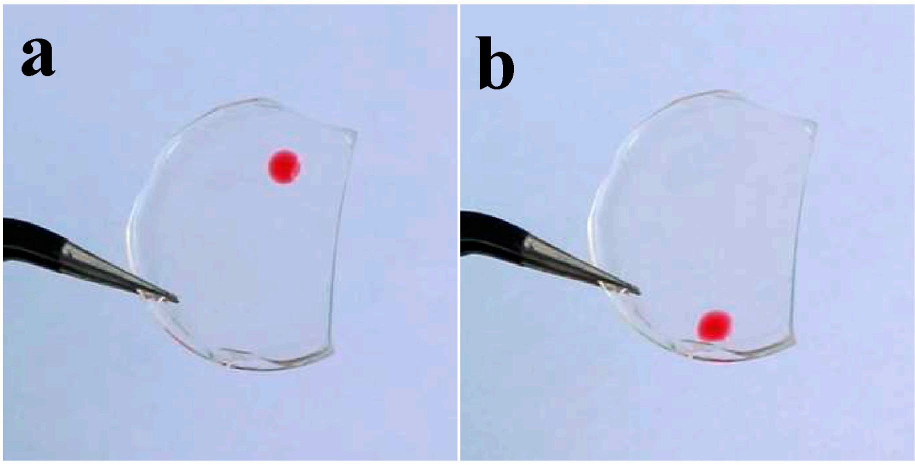

Figure S2. (a) The cured anti-smudge coating was visibly transparent even the coating thickness reached about 1.0 mm. hexadecane droplet could smoothly slid off on above thick coating surface.

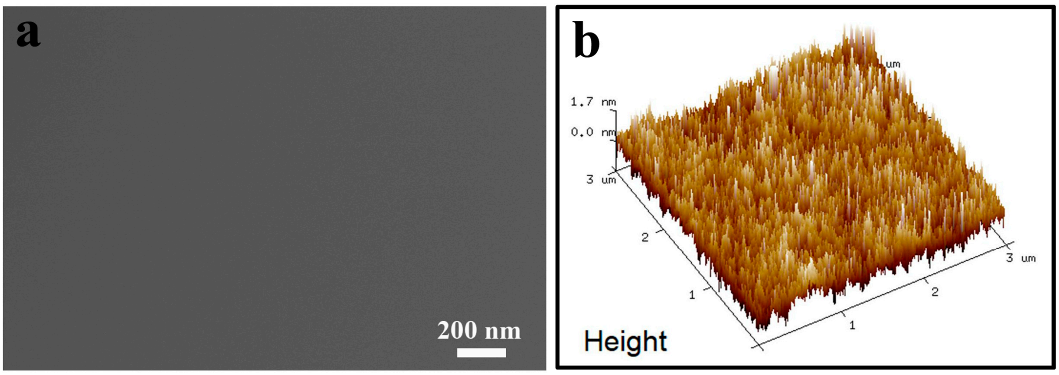

Figure S3. (a) SEM micrograph of the coating surface. (b) AFM 3D topography image of the coating surface. The root-mean-square roughness of the coating surface was measured to be 0.54 nm.

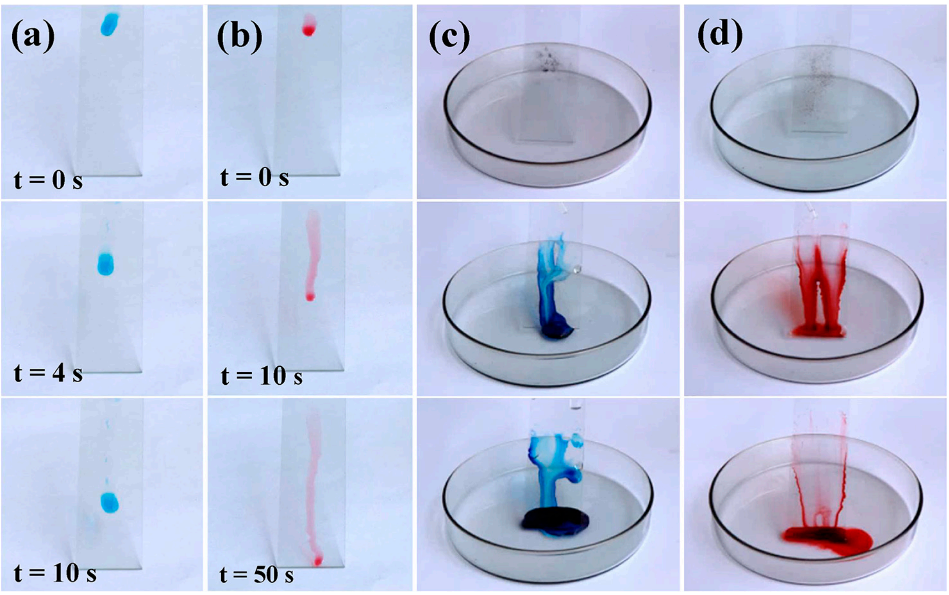

Figure S4. (a) Water droplet spreading and hanging on the uncoated glass plate. (b) Hexadecane droplet spreading on the uncoated glass plate. (c) Dirt (methylene blue powder) scattered on the uncoated glass plate leaving visible blue traces. (d) Dirt (red oil O powder) scattered on the uncoated glass plate leaving noticeable red-dyed paths.

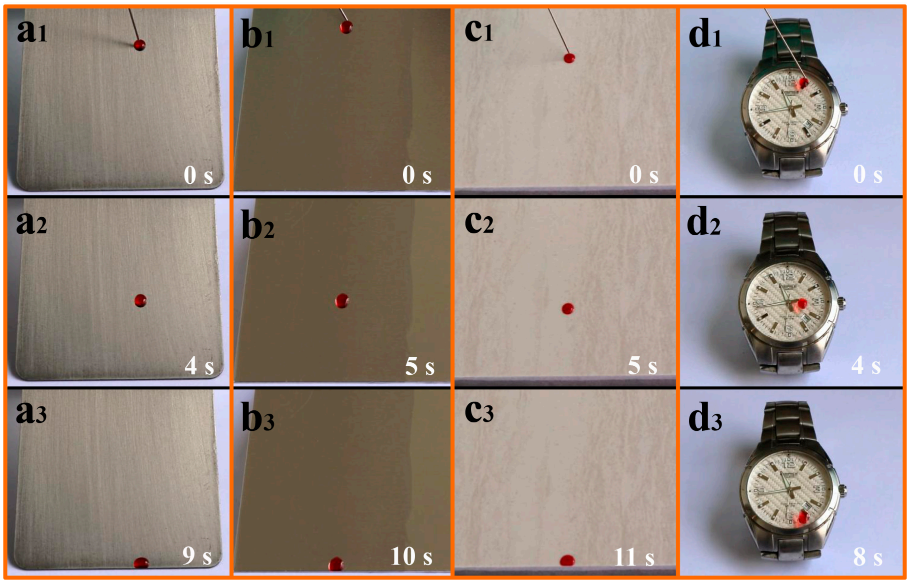

Figure S5. Hexadecane droplets sliding off various coated substrates. (a) A coated steel plate. (b) A half-coated aluminum plate. (c) A coated tile plate. (d) A coated wristwatch.

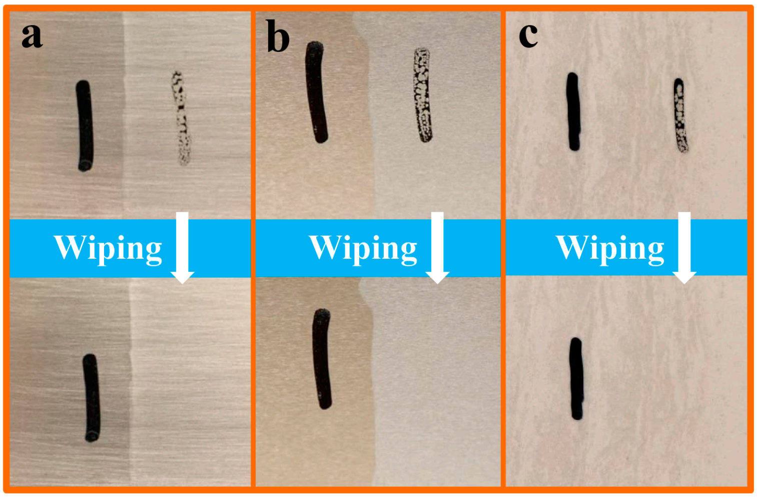

Figure S6. Anti-ink ability demonstrating on various coated substrates. (a) A half-coated steel plate. (b) A half-coated aluminum plate. (c) A half-coated tile plate. The oil-based ink contracted on the coating surface and could be easily wiped off with a tissue.

Table S1. Atomic percentages of the coating surfaces before and after abrasion test

Atom	Atom % Before Abrasion	Atom % After Abrasion
Si	10.15	9.69
O	21.43	20.48
N	8.86	8.99
C	59.55	60.84

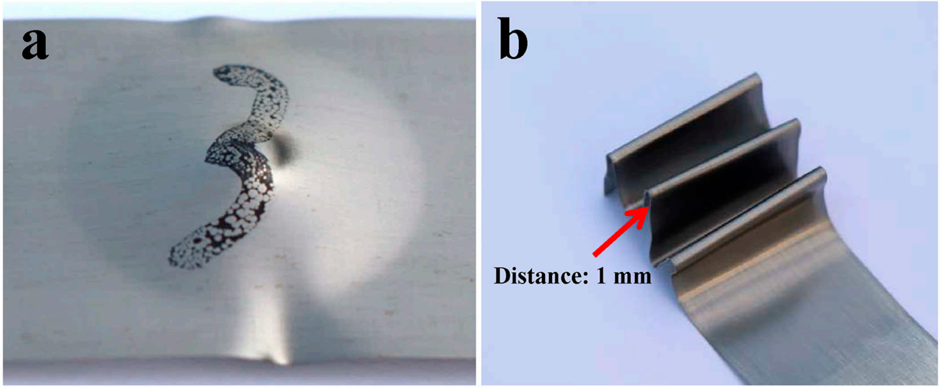

Figure S7. (a) Opposite side of a coated tin plate after suffering from an impact experiment. And the impact test had little influence on the ink repellency of the coating. (b) Photography of a coated tin plate subjected to bending.
